# *N*,*N*′-Bis(1,3-thia­zol-2-yl)methyl­ene­diamine

**DOI:** 10.1107/S1600536811047659

**Published:** 2011-11-16

**Authors:** Farshid Salimi, Hajar Sahebalzamani, Masume Maleki, Behrouz Notash

**Affiliations:** a Department of Chemistry, Faculty of Science, Ardabil Branch, Islamic Azad University, Ardabil, Iran; b Department of Chemistry, Shahid Beheshti University, G. C., Evin, Tehran 1983963113, Iran

## Abstract

In the title compound, C_7_H_8_N_4_S_2_, the dihedral angle between the thia­zoline rings is 71.25 (13)°. In the crystal, inter­molecular N—H⋯N hydrogen bonds connect the mol­ecules into zigzag chains parallel to the *ab* plane.

## Related literature

For applications of thia­zole compounds see: Raman *et al.* (2000 ▶); Karimian (2009 ▶); Shi *et al.* (1996 ▶). For related structures containing an amino­thia­zole moiety, see: Odabaşoğlu & Büyükgüngör, (2006 ▶); Zhao *et al.* (2006 ▶).
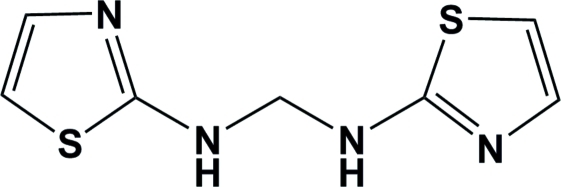


## Experimental

### 

#### Crystal data

C_7_H_8_N_4_S_2_
*M* *_r_* = 212.31
Monoclinic, 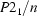

*a* = 7.8598 (16) Å
*b* = 8.9291 (18) Å
*c* = 13.672 (3) Å
β = 96.39 (3)°
*V* = 953.6 (3) Å^3^
*Z* = 4
Mo *K*α radiation
μ = 0.52 mm^−1^
*T* = 298 K
0.45 × 0.35 × 0.3 mm

#### Data collection

Stoe IPDS 2T diffractometer
7352 measured reflections
2551 independent reflections
1544 reflections with *I* > 2σ(*I*)
*R*_int_ = 0.055

#### Refinement

*R*[*F* ^2^ > 2σ(*F* ^2^)] = 0.059
*wR*(*F* ^2^) = 0.162
*S* = 1.10
2551 reflections
126 parameters
2 restraints
H atoms treated by a mixture of independent and constrained refinement
Δρ_max_ = 0.35 e Å^−3^
Δρ_min_ = −0.33 e Å^−3^

### 

Data collection: *X-AREA* (Stoe & Cie, 2005 ▶); cell refinement: *X-AREA*; data reduction: *X-RED*; program(s) used to solve structure: *SHELXS97* (Sheldrick, 2008 ▶); program(s) used to refine structure: *SHELXL97* (Sheldrick, 2008 ▶); molecular graphics: *ORTEP-3 for Windows* (Farrugia, 1997 ▶); software used to prepare material for publication: *WinGX* (Farrugia, 1999 ▶).

## Supplementary Material

Crystal structure: contains datablock(s) I, global. DOI: 10.1107/S1600536811047659/bt5707sup1.cif

Structure factors: contains datablock(s) I. DOI: 10.1107/S1600536811047659/bt5707Isup2.hkl

Supplementary material file. DOI: 10.1107/S1600536811047659/bt5707Isup3.cml

Additional supplementary materials:  crystallographic information; 3D view; checkCIF report

## Figures and Tables

**Table 1:** Hydrogen-bond geometry (Å, °)

*D*—H⋯*A*	*D*—H	H⋯*A*	*D*⋯*A*	*D*—H⋯*A*
N2—H2*A*⋯N1^i^	0.85 (2)	2.07 (2)	2.918 (4)	171 (4)
N3—H3*A*⋯N4^ii^	0.85 (2)	2.07 (2)	2.919 (3)	179 (4)

Symmetry codes: (i) 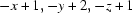; (ii) 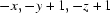.

**Fig. 1.**
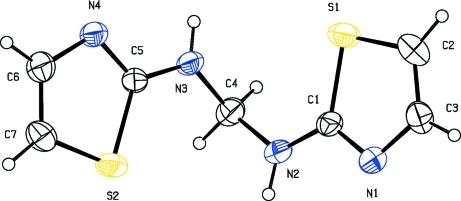
The asymmetric unit of the title compound. Displacement ellipsoids are drawn at the 30% probability level.

**Fig. 2.**
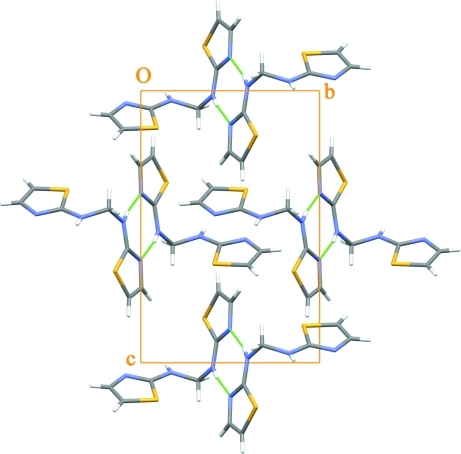
Packing diagram of the title compound. The intermolecular N—H···N hydrogen bonds are shown as green dashed lines.

## supplementary crystallographic information

### Comment

The thiazole ring and its derivatives are of great importance in biological systems due to their vast range of biological activities such as anti-inflammatory, analgesic and antipyretic (Raman *et al.*, 2000; Karimian, 2009), especially against certain breast carcinoma cell lines (Shi *et al.* 1996).

The asymmetric unit of the title compound (Fig. 1) is composed of one *N*,*N*'-bis(2-thiazol-yl)methylenediamin molecule. Bond lengths are in the normal range of thiazole compounds (Odabaşoğlu & Büyükgüngör, 2006; Zhao, *et al.* 2006). The crystal structure is stabilized by intermolecular N—H···N hydrogen bonds, which link the molecules into zigzag chains (Table 1 & Fig. 2).

### Experimental

A mixture of formaldehyde (5 mmol) and 2-aminothiazole (10 mmol) and formic acid (0.88 mmol) was added with stirring at room temperature for 24 hrs.The resulting yellow solid was filtered and washed with cold acetonitrile. Single crystals of the title compound   were obtained by recrystallization of the colorless solid from acetonitrile.

### Refinement

The hydrogen atoms of the N—H groups were found in difference Fourier map and refined isotropically with a distance restraint of N—H 0.850 (19) and 0.850 (18) Å for H2A and H3A, respectively. Hydrogen atoms attached to carbon atoms were positioned geometrically and refined as riding atoms with C—H = 0.93 Å and *U*iso(H) = 1.2 *Ueq*(C) for thiazole rings and C—H = 0.97 Å and *U*iso(H) = 1.2 *Ueq*(C) for methylene group.

### Crystal data

**Table:**

C_7_H_8_N_4_S_2_	*F*(000) = 440
*M**_r_* = 212.31	*D*_x_ = 1.479 Mg m^−^^3^
Monoclinic, *P*2_1_/*n*	Mo *K*α radiation, λ = 0.71073 Å
Hall symbol: -P 2yn	Cell parameters from 2551 reflections
*a* = 7.8598 (16) Å	θ = 2.9–29.2°
*b* = 8.9291 (18) Å	µ = 0.52 mm^−^^1^
*c* = 13.672 (3) Å	*T* = 298 K
β = 96.39 (3)°	Block, colorless
*V* = 953.6 (3) Å^3^	0.45 × 0.35 × 0.3 mm
*Z* = 4	

### Data collection

**Table:**

Stoe IPDS 2T diffractometer	1544 reflections with *I* > 2σ(*I*)
Radiation source: fine-focus sealed tube	*R*_int_ = 0.055
graphite	θ_max_ = 29.2°, θ_min_ = 2.9°
Detector resolution: 0.15 mm pixels mm^-1^	*h* = −10→10
rotation method scans	*k* = −10→12
7352 measured reflections	*l* = −18→16
2551 independent reflections	

### Refinement

**Table:**

Refinement on *F*^2^	Primary atom site location: structure-invariant direct methods
Least-squares matrix: full	Secondary atom site location: difference Fourier map
*R*[*F*^2^ > 2σ(*F*^2^)] = 0.059	Hydrogen site location: inferred from neighbouring sites
*wR*(*F*^2^) = 0.162	H atoms treated by a mixture of independent and constrained refinement
*S* = 1.10	*w* = 1/[σ^2^(*F*_o_^2^) + (0.0781*P*)^2^] where *P* = (*F*_o_^2^ + 2*F*_c_^2^)/3
2551 reflections	(Δ/σ)_max_ < 0.001
126 parameters	Δρ_max_ = 0.35 e Å^−^^3^
2 restraints	Δρ_min_ = −0.33 e Å^−^^3^

### Special details

**Table:**

Geometry. All e.s.d.'s (except the e.s.d. in the dihedral angle between two l.s. planes) are estimated using the full covariance matrix. The cell e.s.d.'s are taken into account individually in the estimation of e.s.d.'s in distances, angles and torsion angles; correlations between e.s.d.'s in cell parameters are only used when they are defined by crystal symmetry. An approximate (isotropic) treatment of cell e.s.d.'s is used for estimating e.s.d.'s involving l.s. planes.
Refinement. Refinement of *F*^2^ against ALL reflections. The weighted *R*-factor *wR* and goodness of fit *S* are based on *F*^2^, conventional *R*-factors *R* are based on *F*, with *F* set to zero for negative *F*^2^. The threshold expression of *F*^2^ > σ(*F*^2^) is used only for calculating *R*-factors(gt) *etc*. and is not relevant to the choice of reflections for refinement. *R*-factors based on *F*^2^ are statistically about twice as large as those based on *F*, and *R*- factors based on ALL data will be even larger.

### Fractional atomic coordinates and isotropic or equivalent isotropic displacement parameters (Å^2^)

**Table:**

	*x*	*y*	*z*	*U*_iso_*/*U*_eq_	
S1	0.17187 (10)	0.85527 (11)	0.66273 (7)	0.0600 (3)	
S2	0.38487 (10)	0.58898 (11)	0.36382 (7)	0.0638 (3)	
N2	0.2786 (3)	0.9024 (3)	0.4824 (2)	0.0534 (7)	
N3	0.1223 (3)	0.6689 (3)	0.4651 (2)	0.0517 (7)	
N4	0.1924 (3)	0.4157 (3)	0.4539 (2)	0.0503 (6)	
C1	0.3071 (4)	0.9249 (3)	0.5804 (2)	0.0458 (7)	
C5	0.2187 (3)	0.5568 (3)	0.4343 (2)	0.0445 (6)	
N1	0.4391 (3)	0.9984 (3)	0.6235 (2)	0.0540 (7)	
C3	0.4335 (4)	1.0032 (4)	0.7244 (3)	0.0609 (9)	
H3	0.5177	1.0521	0.7658	0.073*	
C4	0.1332 (4)	0.8213 (4)	0.4347 (3)	0.0543 (8)	
H4A	0.0294	0.8727	0.4476	0.065*	
H4B	0.1386	0.8234	0.3642	0.065*	
C6	0.3067 (4)	0.3273 (4)	0.4103 (3)	0.0596 (9)	
H6	0.3066	0.2236	0.4161	0.072*	
C2	0.3025 (4)	0.9349 (5)	0.7596 (3)	0.0663 (10)	
H2	0.2841	0.9306	0.8255	0.080*	
C7	0.4177 (4)	0.3987 (4)	0.3592 (3)	0.0637 (9)	
H7	0.5004	0.3523	0.3261	0.076*	
H3A	0.031 (3)	0.645 (4)	0.489 (2)	0.053 (9)*	
H2A	0.358 (4)	0.924 (5)	0.447 (3)	0.084 (13)*	

### Atomic displacement parameters (Å^2^)

**Table:**

	*U*^11^	*U*^22^	*U*^33^	*U*^12^	*U*^13^	*U*^23^
S1	0.0549 (4)	0.0643 (6)	0.0650 (5)	−0.0030 (4)	0.0247 (4)	0.0069 (4)
S2	0.0533 (5)	0.0637 (6)	0.0801 (6)	−0.0071 (4)	0.0333 (4)	−0.0008 (5)
N2	0.0617 (15)	0.0475 (16)	0.0529 (16)	−0.0179 (12)	0.0145 (12)	0.0007 (12)
N3	0.0445 (13)	0.0392 (15)	0.0749 (18)	−0.0073 (10)	0.0228 (12)	−0.0046 (12)
N4	0.0464 (13)	0.0423 (15)	0.0648 (17)	−0.0020 (10)	0.0173 (11)	0.0001 (12)
C1	0.0502 (15)	0.0326 (15)	0.0568 (18)	0.0012 (11)	0.0156 (13)	0.0033 (13)
C5	0.0365 (12)	0.0482 (17)	0.0498 (16)	−0.0050 (11)	0.0098 (11)	−0.0066 (14)
N1	0.0539 (15)	0.0482 (16)	0.0615 (16)	−0.0081 (11)	0.0131 (12)	−0.0041 (13)
C3	0.0624 (19)	0.066 (2)	0.0543 (19)	0.0030 (16)	0.0080 (15)	−0.0124 (16)
C4	0.0527 (16)	0.0458 (18)	0.065 (2)	−0.0034 (13)	0.0072 (14)	0.0001 (15)
C6	0.0561 (16)	0.0466 (19)	0.078 (2)	0.0090 (14)	0.0169 (15)	−0.0049 (17)
C2	0.067 (2)	0.084 (3)	0.0508 (19)	0.0105 (18)	0.0172 (16)	−0.0026 (18)
C7	0.0522 (17)	0.067 (2)	0.075 (2)	0.0116 (15)	0.0218 (16)	−0.0061 (18)

### Geometric parameters (Å, °)

**Table:**

S1—C2	1.735 (4)	N4—C6	1.380 (4)
S1—C1	1.746 (3)	C1—N1	1.311 (4)
S2—C7	1.721 (4)	N1—C3	1.385 (5)
S2—C5	1.731 (3)	C3—C2	1.332 (5)
N2—C1	1.349 (4)	C3—H3	0.9300
N2—C4	1.446 (4)	C4—H4A	0.9700
N2—H2A	0.850 (19)	C4—H4B	0.9700
N3—C5	1.351 (4)	C6—C7	1.338 (5)
N3—C4	1.428 (4)	C6—H6	0.9300
N3—H3A	0.850 (18)	C2—H2	0.9300
N4—C5	1.309 (4)	C7—H7	0.9300
			
C2—S1—C1	89.70 (17)	C2—C3—H3	121.4
C7—S2—C5	88.96 (16)	N1—C3—H3	121.4
C1—N2—C4	123.9 (3)	N3—C4—N2	114.6 (3)
C1—N2—H2A	118 (3)	N3—C4—H4A	108.6
C4—N2—H2A	117 (3)	N2—C4—H4A	108.6
C5—N3—C4	124.1 (3)	N3—C4—H4B	108.6
C5—N3—H3A	117 (2)	N2—C4—H4B	108.6
C4—N3—H3A	116 (2)	H4A—C4—H4B	107.6
C5—N4—C6	109.7 (3)	C7—C6—N4	116.5 (3)
N1—C1—N2	123.8 (3)	C7—C6—H6	121.7
N1—C1—S1	113.4 (2)	N4—C6—H6	121.7
N2—C1—S1	122.8 (2)	C3—C2—S1	109.1 (3)
N4—C5—N3	122.8 (2)	C3—C2—H2	125.4
N4—C5—S2	114.8 (2)	S1—C2—H2	125.4
N3—C5—S2	122.3 (2)	C6—C7—S2	110.0 (2)
C1—N1—C3	110.6 (3)	C6—C7—H7	125.0
C2—C3—N1	117.2 (3)	S2—C7—H7	125.0
			
C4—N2—C1—N1	179.9 (3)	N2—C1—N1—C3	179.9 (3)
C4—N2—C1—S1	1.1 (4)	S1—C1—N1—C3	−1.1 (3)
C2—S1—C1—N1	1.1 (3)	C1—N1—C3—C2	0.5 (5)
C2—S1—C1—N2	−179.9 (3)	C5—N3—C4—N2	−77.4 (4)
C6—N4—C5—N3	178.0 (3)	C1—N2—C4—N3	−60.5 (4)
C6—N4—C5—S2	−0.9 (4)	C5—N4—C6—C7	0.3 (5)
C4—N3—C5—N4	−171.6 (3)	N1—C3—C2—S1	0.3 (4)
C4—N3—C5—S2	7.2 (5)	C1—S1—C2—C3	−0.8 (3)
C7—S2—C5—N4	1.0 (3)	N4—C6—C7—S2	0.4 (5)
C7—S2—C5—N3	−177.9 (3)	C5—S2—C7—C6	−0.8 (3)

### Hydrogen-bond geometry (Å, °)

**Table:**

*D*—H···*A*	*D*—H	H···*A*	*D*···*A*	*D*—H···*A*
N2—H2A···N1^i^	0.85 (2)	2.07 (2)	2.918 (4)	171 (4)
N3—H3A···N4^ii^	0.85 (2)	2.07 (2)	2.919 (3)	179 (4)

Symmetry codes: (i) −*x*+1, −*y*+2, −*z*+1; (ii) −*x*, −*y*+1, −*z*+1.
